# Editorial

**Published:** 1851-08

**Authors:** 


### THE MEDICAL EXAMINER.

PHILADELPHIA, AUGUST, 1851.

### NOTICE TO CORRESPONDENTS.

The attention of our correspondents is respectfully directed to the fact, that the *business* department of this journal is entirely distinct from the *editorial.* Much time and disappointment will be saved, therefore, if *all* letters connected with the former are directed to the Publishers, Messrs. Lindsay & Blakiston, who have the entire management of the subscription list, mailing of the numbers, and all else connected with the distribution.

Communications, and books for review, should be addressed to the Editors.

---

### SYDENHAM SOCIETY OF LONDON.

We are requested to state, that as it is no longer possible to obtain the entire series of the Society's publications, in consequence of no complete sets remaining, of the works issued for the 1st, 2d, and 3d years, and as the applications are frequent from persons who, having certain of the Society's works, are anxious to obtain the others with which they were originally issued, the Council of the Society have thought it expedient to re-arrange those works which can still be obtained, so that *new members*, by subscribing for any past year, may obtain a set of books that shall be complete without those of any other year; whilst any one who wishes it may still obtain them in the order in which they were issued.

LIST OF THE SOCIETY'S WORKS, IN THE ORDER IN WHICH THEY WERE ISSUED.

*For the First Year*, 1843–4.

"Hecker's Epidemics of the Middle Ages," one vol. 8vo., pp. xx. 380.
"Louis on Phthisis," one vol. 8vo., pp. xxxv., 571.
"Th. Sydenham Opera Omnia," one vol. 8vo., pp. xxx., 668.

*For the Second Year*, 1844–5.

"The Seven Books of Paulus Ægineta," vol. 1, pp. xxviii., 683.
"Observations on Aneurism," one vol. 8vo., pp. xii., 524.
"Simon's Animal Chemistry," vol. 1, 8vo., pp. xx., 360, plate.

*For the Third Year*, 1845–6.

"Simon's Animal Chemistry," vol. II., 8vo., pp. xii., 560, 2 plates.
"Paulus Ægineta," vol. II., 8vo., pp. xi., 511.
"Hasse's Pathology," 8vo., pp. xvi., 400.

*For the Fourth Year*, 1846–7.

"The Works of W. Hewson," in one vol., 8vo., pp. lvi, 360, portrait and 8 plates.
"Dupuytren's Lectures on Diseases and Injuries of Bones," one vol. 8vo., pp. xvi., 459.
"The Works of W. Harvey, M. D.," complete in one vol. 8vo., pp. xcvi., 624.

*For the Fifth Year*, 1847–8.

"Paulus Ægineta," vol. III., pp. viii., 653.
"Feuchtersleben's Medical Psychology," pp. xx., 392.
"Microscopical Researches of Schwann and Schleiden," pp. xx., 268, 6 plates.
"Memoirs of the French Academy of Surgery," pp. x., 293.

*For the Sixth Year*, 1848–9.

"Rhazes on the Small Pox and Measles," 8vo., pp. viii., 212.
"The Genuine Works of Hippocrates, translated from the Greek," vol. I., 8vo. pp. x., 466.
"The Works of Sydenham, translated from the Latin," vol. I., 8vo., pp. cvi., 276.

"Rokitansky's Pathological Anatomy," vol. II. (the first issued), 8vo., pp. 375.

*For the Seventh Year*, 1849–50.

"The Genuine Works of Hippocrates," vol. II., pp. 406.
"Essays on Puerperal Fever, and other Diseases peculiar to Women," 8vo., pp. 552.
"The Works of Sydenham, translated from the Latin," vol. II., pp. 396.

*For the Eighth Year*, 1850–1.

"Rokitansky's Pathological Anatomy," vol. III., 8vo., pp. xvi., 467.
"Hunter on the Gravid Uterus," one vol. folio, 34 Plates, with descriptive letter-press.

*List of the Works that may still be had, arranged in the order in which they may be obtained by New Members, on payment of five dollars for each year.*

*Third Year*, 1845–6.

"Sydenhami Opera Omnia." One vol.
"Hasse's Pathological Anatomy."
"Rhazes on the Small-pox and Measles."

*Fourth Year*, 1846–7.

"The Works of Hewson.'
"Dupuytren's Lectures on Diseases and Injuries of Bones."
"Memoirs of the French Academy of Surgery."

*Fifth Year*, 1847–8.

"Feuchtersleben's Medical Psychology."
"Microscopical Researches of Schwann and Schleiden."
"The Works of W. Harvey, M. D."

*Sixth Year*, 1848–9.

"The Genuine Works of Hippocrates, translated from the Greek." In two vols.
"Essays on Puerperal Fever, and other Diseases peculiar to Women."

*Seventh Year*, 1849–50.

"The Works of Sydenham, translated from the Latin." In two vols.
"Rokitansky's Pathological Anatomy." Vol. II. (the first issued.)

*Eighth Year*, 1850–1.

"Rokitansky's Pathological Anatomy." Vol. III.
"Hunter on the Gravid Uterus." One vol., folio. 34 Plates, with descriptive letter-press.

---

We insert, with much pleasure, the following card from Dr. Gross, and trust that it may receive the prompt attention which its importance demands.

### RESULTS OF SURGICAL OPERATIONS IN MALIGNANT DISEASES.

*To the Medical Profession of the United States:*

The undersigned having been appointed, at the last meeting of the American Medical Association, Chairman of the Committee on the "Results of Surgical Operations in Malignant Diseases," respectfully solicits contributions to the subject, founded upon personal observation. To place the subject in as tangible a form as possible, he begs leave to direct attention to the following points:

1. The difference between cancerous and cancroïd diseases, or those affections which are truly malignant, and those which are only partially so. In the former category are comprised scirrhus, encephaloïd, and melanosis; in the latter, certain maladies of the skin and mucous tissues, as lupus, cheloïd, eiloïd, and cancer of the lip.

2. The precise seat of the disease, as the skin and subcutaneous cellular tissue; the eye, ears, nose, face, lips, tongue, salivary glands, jaws, and gums; the lymphatic ganglions of the neck, axilla, groin, and other regions; the mammary gland, uterus, ovary, vulva and vagina, penis and testis; the anus and rectum; and, finally, the extremities.

3. The age, sex, temperament, residence, and occupation of the patient.

4. The cause of the disease, its progress, and the state of the part and of the system at the time of operation.

5. Mode of operation; whether by the knife, caustic or ligature.

6. Time of death, or relapse, after the operation.

7. Examination of the morbid product; how conducted—whether by the unassisted eye alone, or by means of the microscope, and chemical tests.

The undersigned hopes that the importance of the subject confided to him, as chairman of the committee above referred to, will be sufficiently appreciated by his professional brethren, to induce them to aid him in carrying out the wishes of the American Medical Association. The subject is one of absorbing interest, and cannot fail, if properly treated, to elicit matter of the greatest benefit. It is very necessary that all communications upon the subject should be sent to the chairman of the committee by the 1st of January, 1852.

Medical journals, and newspapers friendly to the interests of medical science, will confer a favor upon the undersigned by inserting the above notice.

S. D. Gross, M. D.

*University of Louisville, June* 29, 1851.

### FOURTH ANNUAL MEETING OF THE AMERICAN MEDICAL ASSOCIATION.

We copy from the Charleston Medical Journal and Review, for July, the following account of the opening of the meeting in Charleston in May last:

It was composed of delegates, many of high distinction, from twenty-one States of our Union, and a more respectable assemblage has seldom been convened on any former occasion. The President, Dr. R. D. Mussey, of Cincinnati, took the chair at 11 o'clock, and extended a cordial salutation to the members upon their re-union, and congratulations upon the many benefits resulting from these annual assemblages of the wisdom and experience from all parts of the Union.

Upon being seated, the Committee of Reception advanced, and, through their chairman, Dr. T. Y. Simons, addressed the President and members in the following terms:

" *Mr. President and Gentlemen:*—It is my pleasing and honorable duty, as Chairman of a Committee representing the South Carolina Medical Association, to express to you the high appreciation of your acceptance of the invitation of that body, and gratification in beholding so many of our medical brethren, of every section of the Union, assembled in Charleston, the ancient metropolis of South Carolina, and to tender to you, with the deepest emotions, our most heartfelt welcome.

" We will cheerfully unite with you in all efforts to elevate the moral and intellectual excellence of our profession, and promote the cause of science and humanity, but beyond this, we desire to extend to you, individually and collectively, all the social hospitalities of which we are susceptible.

" The assembly of so large a number of medical gentlemen from all parts of the Union, holding eminent positions in the profession, coming, in many instances, from a great distance, at considerable expense, inconvenience and personal sacrifice, for the promotion of medical improvement, and thus likewise subserving the cause of science and humanity, is, in my opinion, a sublime spectacle. We seek no honors nor trophies; we crave no ambition, pomp, nor self-aggrandisement; we desire only, and if we succeed the reward is ample, to do good to mankind. We unite with you as brothers, in these noble and benevolent efforts, and we invoke, on this solemn occasion, the blessings of the Great Supreme.

" Mr. President and gentlemen, I shall not detain you, except again to repeat, that with our warmest feelings we bid you a most hearty welcome."

To which Dr. Mussey replied substantially as follows:

"*Mr. Chairman and Gentlemen of the Committee:* It is with no ordinary feelings that I respond in behalf of the American Medical Association, and express our gratification at your warm and hospitable reception.

"Charleston has been long renowned for the hospitality of her citizens, and the enlightened character of her physicians, both as regards moral and intellectual endowments. High, however, as she stood, I was not aware until I arrived here, that the Medical Society of South Carolina was the first body that proposed that means should be adopted for the improvement of medical education. I now, Mr. Chairman, return you our thanks for your kind, and hospitable, and warm reception; and feel confident that we will harmoniously unite in the promotion of the grand objects of the Association."

The Committee on Nominations having reported, through their chairman, suitable candidates for the officers of the Association for the ensuing year, the same was confirmed, and Dr. James Moultrie, of South Carolina, was declared President of the Association.

The President elect then took the chair, and returned thanks for the honor thus conferred upon him, in the following terms:

"*Gentlemen of the Association:* I know not in what terms suitably to express my sincere acknowledgments for this unlooked for manifestation of appreciation and kindness.

"When I look around, and recognize the many superior intellects and higher professional acquirements by which I am surrounded, I feel that I am about to occupy a position which more rightfully belongs to another.

"But, if a life devoted to the interests of the profession, and the promotion of the objects for which this institution is founded, give me any claim to consideration, I have the assurance that the mantle of my illustrious predecessors may not be unworthily or ungracefully worn.

"In the language of the first of them, I have ever 'loved my profession;" nay more, I am proud of it. I have loved it for its sympathies and suavities; its benignities and charities; its activities and self-sacrifices; for the boundless scope of knowledge which is opened before it; its disinterested pursuit of the true, the beautiful, and the good; the exalted mission which has been appointed to it on earth; and the universal appreciation in which it is held, and confidence reposed in it, by mankind; and I am proud of it, not alone for what has thus been adverted to, but for its habitual singleness of purpose; its almost immunity from prison discipline; the little notoriety it enjoys in the statistics of crime; and the little trouble it has given to the tribunals of justice, or of civil jurisprudence.

"Never, however, have I been so proud of it as when, four years ago, I enjoyed the gratification of meeting so large an assemblage, in the city of 'Brotherly Love,' characterized by so much intelligence, so much urbanity, so much dignity, and so much condescension; convened for the purpose of systematizing the principles of professional prosperity, and cultivating those habits of social harmony, the practice of which have immemorially obtained for it the definite epithet of 'the paternity:' or when, two years afterwards, at the modern abode of the 'Humanities,' its labors of usefulness and love were renewed, amidst the outpourings of hospitalities, which could only have issued from heads and hearts the most highly cultivated and refined.

"The accidents of life, and the visitations of Providence, which come unto all of us in turn, I have to regret, denied me the gratification of being also present, on the intermediate occasions, in the great valley of the Mississippi, and the commercial emporium of the Chesapeake.

"For the privilege, as well as the distinction, of presiding over a body constituted of such elements, I am profoundly grateful. I thank you, gentlemen, again and again; and when our present labors shall have closed, and we shall have once more reached our respective homes, I hope we shall be able to realize, in our experience, that 'it was good for us,' in like manner, 'to have been here.'"

The following is a correct list of the number of States represented, and the number of delegates from each:

| | | | | | | | |
|---|---|---|---|---|---|---|---|
| 1. | Massachusetts, | 10 | delegates. | 14. | Louisiana, | 2 | delegates. |
| 2. | Rhode Island, | 5 | " | 15. | Tennessee, | 3 | " |
| 3. | Connecticut, | 3 | " | 16. | Kentucky, | 4 | " |
| 4. | New York, | 17 | " | 17. | Ohio, | 5 | " |
| 5. | New Jersey, | 6 | " | 18. | Indiana, | 1 | " |
| 6. | Pennsylvania, | 35 | " | 19. | Illinois, | 1 | " |
| 7. | Delaware, | 2 | " | 20. | Missouri, | 3 | " |
| 8. | Maryland, | 5 | " | 21. | Dist. of Columbia, | 2 | " |
| 9. | Virginia, | 11 | " | | | | |
| 10. | North Carolina, | 7 | " | | Total, | 185 | |
| 11. | South Carolina, | 38 | " | | Permanent members, | 10 | |
| 12. | Georgia, | 23 | " | | | | |
| 13. | Alabama, | 1 | " | | Grand Total, | 195 | |

